# Supplementary material for: The influence of random number generation in dissipative particle dynamics simulations using a cryptographic hash function
Source: PLoS One. 2021 Apr 27;16(4):e0250593. doi: 10.1371/journal.pone.0250593 (PMC8078758; doi:10.1371/journal.pone.0250593)
Supplement: S1 File — (PDF) [file pone.0250593.s001.pdf]

The influence of random number generation in dissipative particle dynamics simulations using a cryptographic hash function

Kiyoshiro Okada, Paul E. Brumby, Kenji Yasuoka*,

Department of Mechanical Engineering, Keio University,Yokohama, Kanagawa, Japan

* yasuoka@mech.keio.ac.jp

S1 File

These are the results of DPD-water system using uniform random numbers not gaussian random numbers for random forces.

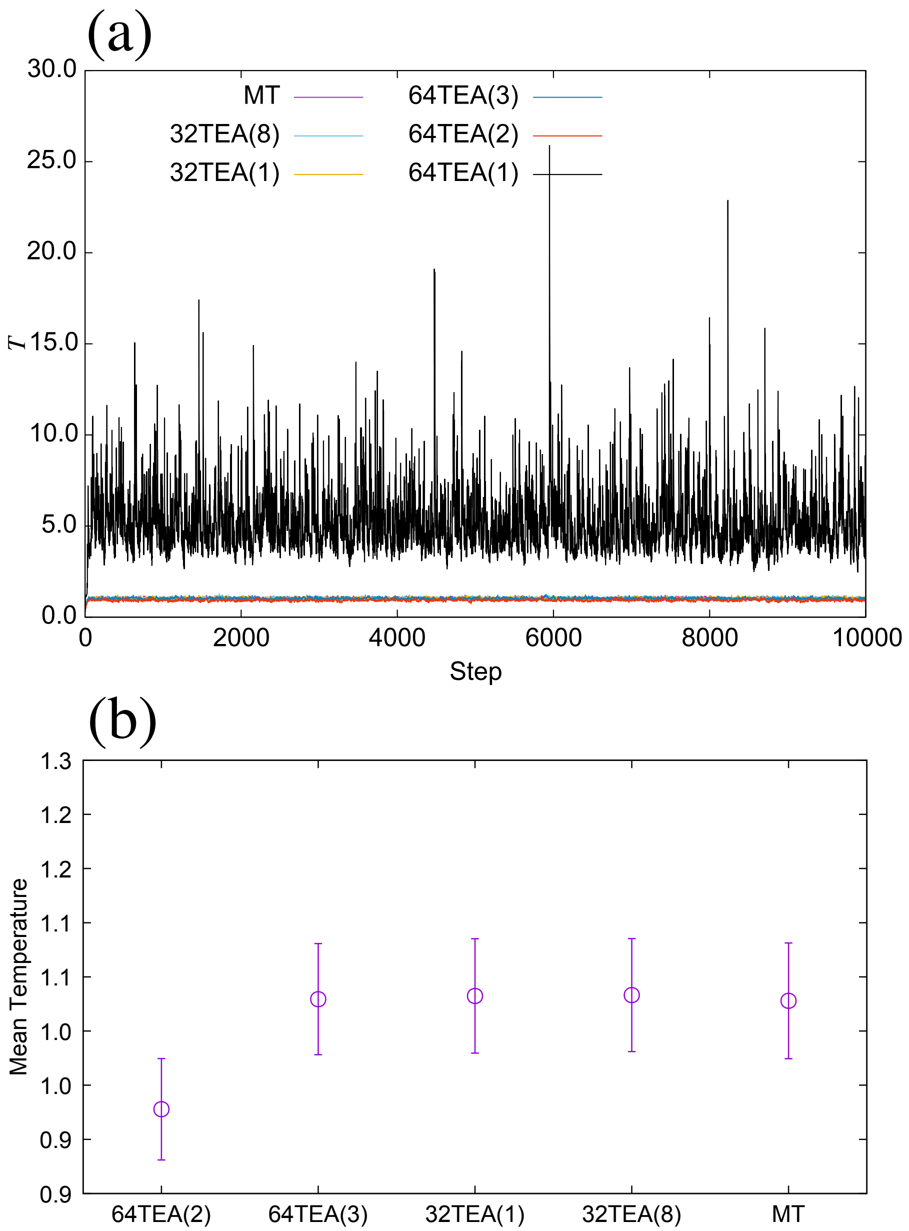

Fig 1. (a)Temperature for the water system,(b)Mean Temperature and its fluctuations.

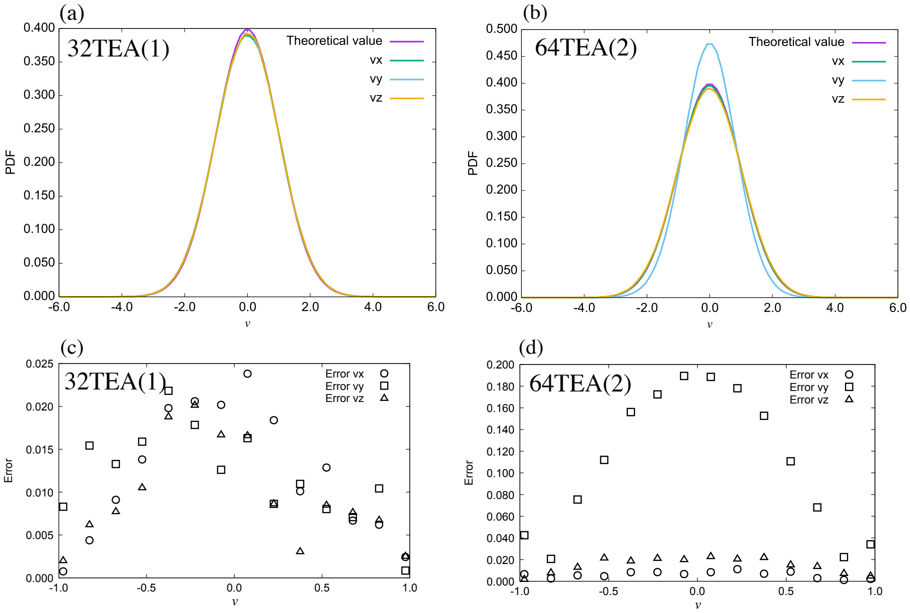

Fig 2. Probability distribution function (PDF) of the velocity for water system by (a) 32TEA(1) and by (b) 64TEA(2) and the error rate of velocity distribution by (c) 32TEA(1) and by (b) 64TEA(2).

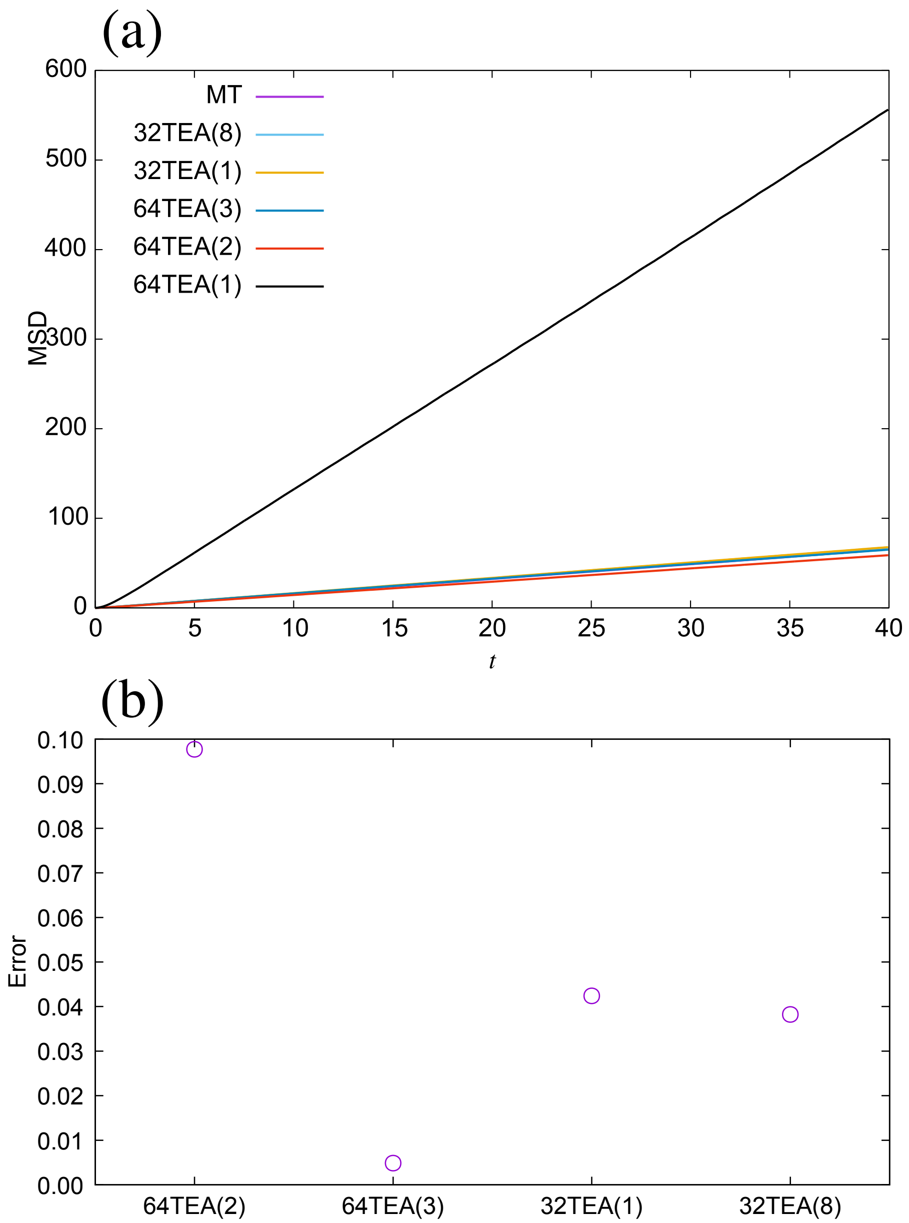

Fig 3. (a) Mean square displacement for water system, (b) Error rate of diffusion coefficient.

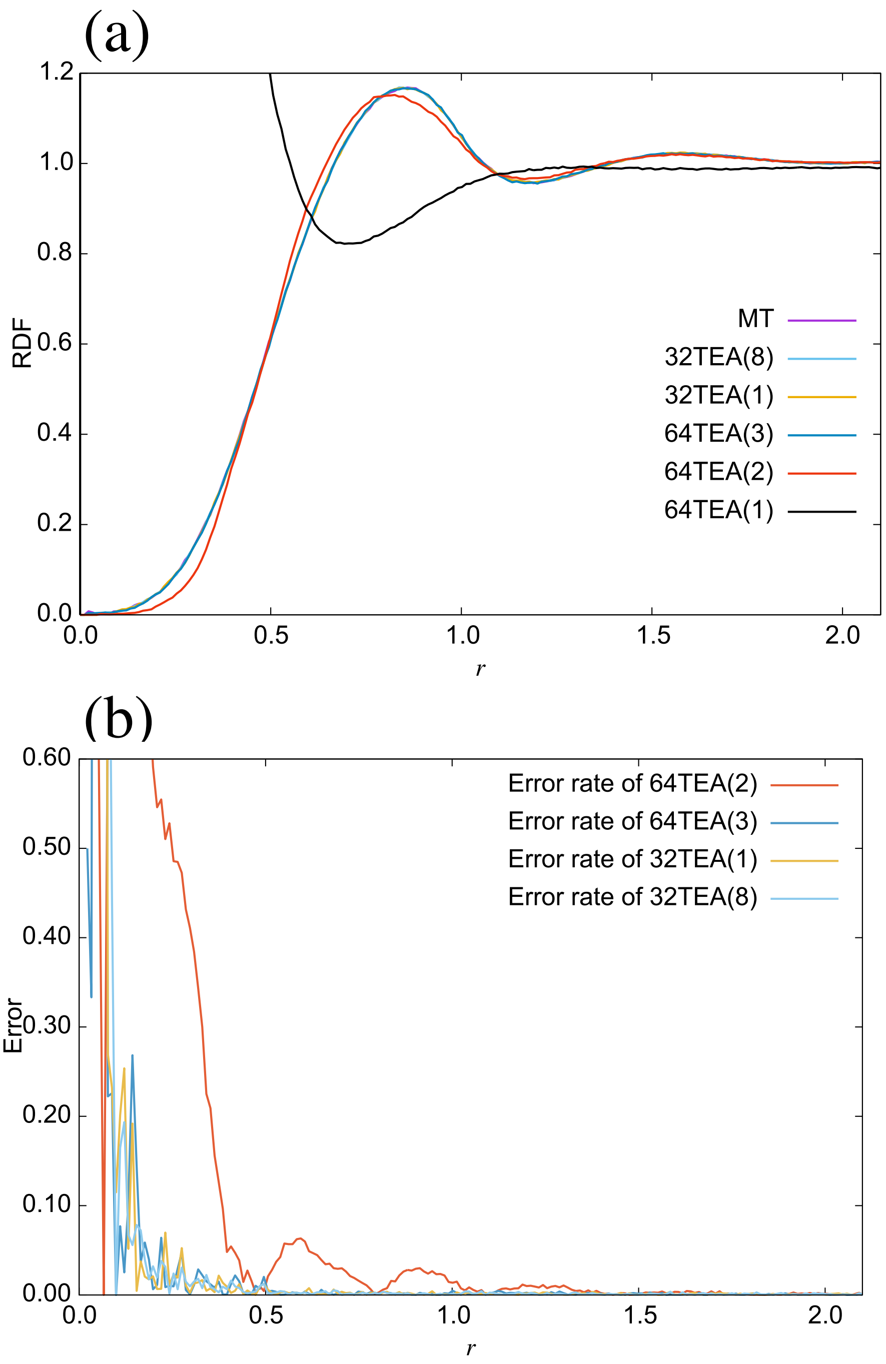

Fig 4. (a) Radial distribution function, (b) Error rate of Radial distribution function.
