# Supplementary material for: The influence of random number generation in dissipative particle dynamics simulations using a cryptographic hash function
Source: PLoS One. 2021 Apr 27;16(4):e0250593. doi: 10.1371/journal.pone.0250593 (PMC8078758; doi:10.1371/journal.pone.0250593)
Supplement: Attachment — Submitted filename: Response to Reviewers.pdf [file pone.0250593.s002.pdf]

The Editors,

PLOS ONE

March 23, 2021

Dear Reviewers

Thank you for reading our paper and for taking the time to give us your kind feedback.

Based on the your comments and questions, we have performed additional validation tests and have updated the manuscript. Please find our written responses to your questions below.

For Reviewer #1:

Thank you very much for your kind response. We have done some additional testing based on your feedback.

1. For the modified pseudo-RNGs to be adopted for other systems than the test case, the authors need more rigorous tests than the current tests on a rather small set of generated random numbers (9500), which is well below the prime period of the RNGs. See for example Ref. 45 for the commonly used tests for PRNGs.

In the first version of our manuscript the random number test described was performed to narrow down certain properties to see how they affect the DPD simulation results. However, as you pointed out, in addition to this, a more general random number test would be much more appropriate. Following your recommendation we have performed further tests of randomness, this time using the NIST test set. As a result of applying the NIST verification tests to 32TEA, which is the one we recommended in this manuscript, surprisingly, we found that the tests were all passed when using just one round. We have also confirmed that 32TEA(2), 32TEA(3), 32TEA(4), 32TEA(5), 32TEA(6), 32TEA(7), 32TEA(8), and 32TEA(32), similarly pass all 15 NIST tests. (Page 9 : Results and discussion) This represents a significant improvement in randomness when compared to a previous work (Ref. 31: Zafar F, Olano M, Curtis A. GPU random numbers via the tiny encryption algorithm. In: Proceedings of the Conference on High Performance Graphics. Citeseer; 2010. p. 133–141.) which was not able to fully pass all of the NIST tests. We believe that these results show that, on balance, 32TEA(1) is the best choice for use in DPD simulations. Thank you for your insightful comments which have really helped us to improve the standard of this manuscript.

2. The finding that 32TEA(1) is the optimal algorithm in terms of randomness is indeed interesting. Can the authors attempt to explain why this might be the case, otherwise it is difficult to envision if the 32TEA(1) would work as well for other use cases beyond the test random number set and the particular simulation composed of 270 coarse-grained water molecules at liquid state. Also in Fig 2, for 32TEA algorithms only data for n=1 and n=8 are shown. What are the results for other values of n for 32TEA?

We believe that the 32TEA with only a single round as used in our work produces numbers with a higher degree of randomness because of our choice of using the distance between particles as a seed. As mentioned above, we have run additional randomness checks using the NIST test set and the 32TEA with a single round passed all tests, unlike in previous studies. The difference between our work and these previous studies is in our choice of seed number. This indicates that if the seed number if chosen which already has a high degree of randomness, then 32TEA(1) can generate a superior quality of random numbers while also being less computationally demanding.

Following your very helpful comments we have also simulated a biomembrane system so as to have an additional test case with which to demonstrate the the wider applicability of our findings. The results from these DPD simulations for 32TEA(1) and 32TEA(32) revealed no difference in the properties obtained between the two algorithms. We hope that this helps to demonstrate the wider applicability of the 32TEA(1) for DPD simulations. (Page 14, 15, 16 : Results and discussion)

About the results for other values of n for 32TEA, only the minimum number of rounds required to pass each test is shown because we have confirmed that the same requirements are satisfied when using a higher number of rounds.

3. A related study for implementing and comparing the effects of different PRNGs including TEA(8) for parallel DPD simulations (along the line with Phillips el al (Ref 43), and Afshar et al (ref 45)) where more quantities and systems, that are typical of DPD studies, were considered is Nguyen and Plimpton, Computational Materials Science 2015, 100, 173-180.

Thank you for highlighting a work which is of great relevance here. We updated the manuscript to include this citation in our introduction. (Page 2 : Introduction)

For Reviewer #2

Thank you very much for your kind response. For the random numbers obtained using the 32TEA, we have performed additional tests using the commonly adopted NIST test set. In this way, we have shown that 32TEA(1) is able to generated numbers of a sufficiently high randomness for general use in DPD simulations. (Page 9 : Results and discussion) We have also compared 32TEA(1) with 32TEA(32) for more complex systems using biological membranes as well as water. As a result, we could confirmed that there was no difference in the physical properties obtained using either algorithm. This shows that the 32TEA(1) is clearly the optimal choice due to its lower computational cost. (Page 14, 15, 16 : Results and discussion)

1. Page 1: DPD simulations allow for the use of longer time-steps when compared with many other CG approaches, such as the MARTINI force-field. Here, you mentioned that MARTINI force field, rather than an approach. Hence, it is an inaccurate statement.

Thank you so much for bringing this to our attention. We have corrected the text to fix this mistake. (Page 1 : Introduction)

2. Pages 3-4: There is no need to discuss DPD fundamentals in details. It has been done in a lot of publications.

Thank you for your suggestion. We have removed these unnecessary parts from the manuscript and revised the text to make it simpler. (Page 1,2 : Introduction)

Information of all authors:

Kiyoshiro Okada[1] (kiyoshirookada@keio.jp)

Paul Edward Brumby[1] (p.brumby@keio.jp)

Kenji Yasuoka[1] (corresponding author, yasuoka@mech.keio.ac.jp)

[1]Department of Mechanical Engineering, Keio University, 3-14-1 Hiyoshi, Kohoku-ku, Yokohama 223-8522, Japan.

Tel: +81-45-566-1523

Fax: +81-45-566-1495

We look forward to hearing from you at your earliest convenience.

Yours sincerely,

Kenji Yasuoka
